# Extent of craniofacial fungal osteomyelitis in a ketoacidotic patient may predict optic nerve involvement: A case report

**DOI:** 10.1016/j.ijscr.2022.107299

**Published:** 2022-06-13

**Authors:** Jennifer Adams, Khalid N. Sheikh, Adam Bender-Heine

**Affiliations:** a School of Medicine, University of Texas Rio Grande Valley, United States of America; b Department of Head and Neck Surgery, Facial Plastic and Reconstructive Surgery, University of Texas Rio Grande Valley, United States of America

**Keywords:** Fungal osteomyelitis, Mucormycosis, Opportunistic infection, Diabetes mellitus, Otorhinolaryngology, Case report

## Abstract

**Introduction:**

Mucormycosis is an opportunistic mycosis common in poorly-controlled insulin dependent diabetic patients particularly with ketoacidosis. Fungal osteomyelitis is a life-threatening condition affectation of the nose and paranasal sinuses within the orofacial region.

**Presentation of case:**

We present a 63-year-old diabetic male patient with maxillary mucormycotic osteomyelitis threatening his better seeing eye and review the clinical symptoms, relevant imaging, and management.

**Discussion:**

We highlight a rare pattern of craniofacial fungal bone infection with maxillary and orbital involvement that ultimately spared the optic nerve. This case report offers the clinician a review of important clinical and diagnostic findings that can help direct the need for orbital exenteration.

**Conclusion:**

Maxillary mucormycotic osteomyelitis is an aggressive infection that needs to be addressed promptly to prevent fatal consequences.

## Highlights

• Fungal maxillary osteomyelitis must be addressed immediately to avoid compromise of cranial nerves and loss of vision.
• An understanding of MRI findings suggesting Mucor orbital involvement is essential to monitoring osteomyelitis progression.
• Sparing the orbit near the better-seeing eye is critical to post-recovery quality of life.

## 1 Introduction

Mucormycosis is an opportunistic mycosis common in immunocompromised patients, especially in poorly controlled insulin-dependent diabetic patients [1]. Risk factors that increase the risk of mucormycosis include any immunosuppression including advanced HIV infection, leukemias, lymphoma, systemic treatment with corticosteroids, and severe burns [2]. Upon entering the tissues, spores infiltrate blood vessels, inducing an inflammatory reaction, which leads to thromboses and ischemic necrosis. Osteomyelitis of the facial skeleton is a rare condition. It uncommonly occurs in the maxilla, which has significant collateral blood flow, thin cortical bones which make it less prone to infection [3]. Direct culture of nasal discharge, bone, or tissue will reveal broad hyphae with uneven thickness, irregular branching and septations [4].

The most advanced and life-threatening clinical form of opportunistic mycosis in patients with Diabetes Mellitus (DM) is rhinocerebral mucormycosis, which involves rapid progression with invasion of the sinuses, eyes, cranial bones, and brain [5]. Invasive fungal sinusitis typically starts intranasally involving the turbinates, which form the medial wall of the maxilla. Bony invasion precedes neural invasion, beginning in the paranasal sinuses and spreads along infected vessels to the retroorbital tissues and cavernous sinus. Clinical findings may include proptosis, ophthalmoplegia, and edema of the lids and retina and then hemorrhagic infarction when spread to brain parenchyma [6]. We present a review from our experience of Mucor of the maxilla causing extensive bony involvement and necrosis in a male with uncontrolled diabetes. This work has been reported in line with the SCARE 2020 criteria [7].

## 2 Case presentation

**Fig. 1:**
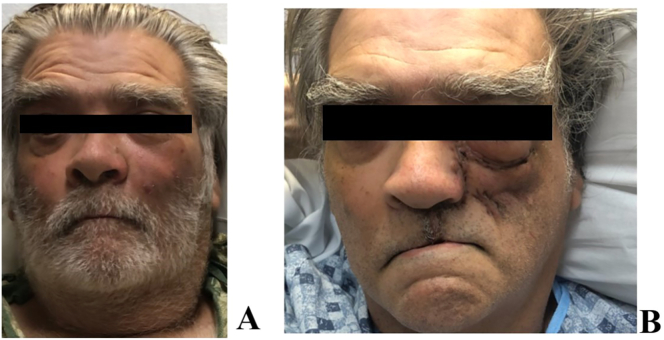
(A) Preoperative picture of patient with left cheek skin ecchymosis and left scleral chemosis. (B) Postoperative week 2.

**Fig. 2:**
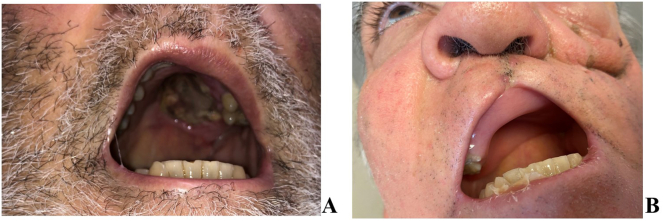
(A) Preoperative palatal necrotic lesions; (B) postoperative palatal defect.

A 63 year-old man presented to the Emergency Department in ketoacidosis with an ulcerating, necrotic defect of the left hard palate with left facial ecchymosis and left scleral chemosis with extraocular muscles and diplopia (Figs. 1A, 2A).

Patient reports no medication use. Family and psychosocial history is noncontributory.

**Fig. 3:**
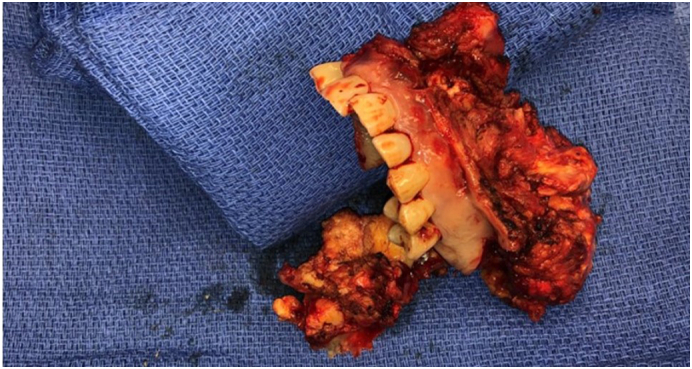
Resected maxillary specimen

After soft tissue and bone biopsy confirmed mucormycosis, the patient underwent a left infrastructure maxillectomy with orbital sparing and two subsequent debridements. Extensive resection of the left zygomatic arch, left hard and soft palate and the surrounding soft tissues was performed (Fig. 3). During the procedure, severe involvement and necrosis of the left side lateral nasal wall, floor, and septum were visualized. Osteolysis involving the bony walls of the left maxillary sinus, portions of the bony walls of the left paranasal sinuses, left bony orbit, the inferior aspect of the orbital floor and left greater and lesser sphenoid wings was apparent.

Surgical resection resulted in a large hard palate defect with an oral-nasal-maxillary continuity. Surgery was performed by the Head and Neck surgeon on call. Post-operative magnetic resonance imaging (MRI) revealed residual enhancement of the inferior rectus muscle as well as subtle asymmetric enhancement along the optic nerve sheath on the left compared to the right. as increased caliber with signal intensity changes within the nerve.

Pathological exam of intraoperative specimens identified the presence of broad nonseptate hyphae under special stains such as hematoxylin and eosin (H and E), Periodic acid–Schiff (PAS). These features were consistent with deep fungal invasion of bone. In the inpatient setting, the patient was given an infusion of isotonic saline with dextrose to stabilize cardiovascular status and 20 milliequivalents/ h of IV potassium chloride with IV regular insulin for management of the ketoacidosis. The patient had a gastrostomy tube placed and was discharged with subsequent outpatient treatment including three months of Amphotericin B and improved diabetic management. Post-surgically he was fitted with a palate obturator which was well tolerated and allowed normal speech and nutrition by mouth.

## 3 Discussion

The most common clinical presentation of mucormycosis is nasal and paranasal sinus infection which begins with inhalation of spores into the paranasal sinuses of a susceptible host [6]. Symptoms vary and can include worsening vision and diplopia. More advanced features include chemosis, proptosis, ophthalmoplegia, optic atrophy, and blindness. Bone involvement in the orofacial region manifestations includes rhinorrhea, facial cellulitis, nasal discharge, and turbinate necrosis along with lethargy fever and headache. The two main factors predisposing to osteomyelitis of the maxilla include dental infections, maxillary sinusitis, trauma, and radiation [3]. In this case, the main risk factor was poorly controlled diabetes mellitus and fungal infection. The patient had history recurrent maxillary sinusitis. Approximately 70 % of cases of maxillary osteomyelitis are related to diabetes mellitus as hyperglycemia alters the blood flow distribution to the maxilla [3].

Recent COVID-related manifestations of maxillary mucormycosis osteomyelitis have a distinct pattern of facial bone involvement similar to that of our case. One case series reported 14 patients with comorbid COVID and diabetes presented with various mucor involvement of the maxilla, zygoma, and orbital rim [8]. Another similar case demonstrated mucormycotic osteomyelitis of the nasomaxillary-zygomatic complex following trauma in a middle-aged man [9]. Our case is unique in that our patient presented with severe mucormycotic maxillary osteomyelitis with inferior orbital rim involvement sparing the optic nerve without history of trauma or reported COVID infection.

Radiography may be useful as a supportive diagnostic aid rather than being a definitive diagnostic modality. The anatomical extent of (rhino-oculo-cerebral mucormycosis) ROCM has been classified into stage I (limited to the nasal mucosa), stage II (paranasal sinus extension), stage III (orbital extension), and stage IV (extension to central nervous system) [10]. Patients with Stage III disease may undergo orbital exenteration to avert cerebral extension. Globe involvement is suggested by thickening and enhancement of any of the three coating layers of the globe on MRI [11]. Bone involvement, especially orbital integrity, may be predictive of optic nerve involvement in upstaging from II to III. More studies are needed to develop a classification for prognostic imaging factors that account for bony involvement.

This patient in the case was originally thought to have compromise of the optic nerve based on imaging findings and presentation. There was indication of increased caliber with signal intensity changes within the nerve suggesting direct invasion of the optic nerve on MR imaging, which supported the indication for total exenteration. Optic nerve involvement can be due to arterial or venous occlusion, nerve infarction, or direct infiltration of the optic nerve. Infarction is seen as high-signal intensity of the nerve on diffusion-weighted imaging [12].

Even if the orbit can be spared, patients with advanced disease are often still left with a major mid-face deformity following maxillectomy which impairs normal speech and swallow. Reconstructive options include prosthetic obturators and bone transfer. A prosthetic palatal obturator is a simple and inexpensive post-surgical restorative option [12]. The advantages of the palatal obturator include acceptable cosmesis, cost-efficiency, relative ease of manufacture, facilitation of disease surveillance, deferred surgery in elderly patients or those with poor health. The options for microvascular bone free tissue transfer include scapula and fibula [12]. Autologous bone transfer helps restore and support facial contours while also allowing for near normal restoration of speech and swallow. The disadvantages of free tissue transfer include need for prolonged surgery and highly technical surgical expertise. In this case, the patient had sufficient remnant native dentition and access to a low-cost prosthetic expert that was available to fit the patient for an obturator. The patient was able to regain normal speech and swallow with the obturator. There was, however, some visible facial deformity from the loss of volume which was not restored.

## 4 Conclusion

Immunocompromised patients are vulnerable to fatal fungal infections involving the bones and soft tissues of the oral cavity. Treatment should be undertaken as soon as the diagnosis of rhino-orbital mucormycosis is suspected to limit progression of disease and resulting facial disfiguration as well as possible death. Sparing the orbit particularly in the case of disease near the better or only-seeing eye is critical to post recovery quality of life. In this case report we have highlighted these important MRI findings in the case of patient a with facial osteomyelitis that had mild to moderate orbital findings but ultimately was successfully treated without compromise to his vision.

## Patient perspective

N/A.

## Informed consent

Written informed consent was obtained from the patient for publication of this case report and accompanying images. A copy of the written consent is available for review by the Editor-in-Chief of this journal on request.

## Provenance and peer review

Not commissioned, externally peer-reviewed.

## Ethical approval

N/A.

## Funding

This research did not receive any specific grant from funding agencies in the public, commercial, or not-for-profit sectors.

## Guarantor

Adam Bender-Heine M.D.

## Research registration number

N/A.

## CRediT authorship contribution statement

JA was involved in the writing of the manuscript. ABH was involved in the editing/supervision of the manuscript.

## Declaration of competing interest

None.
